# High-Resolution 3D MRI With Deep Generative Networks via Novel Slice-Profile Transformation Super-Resolution

**DOI:** 10.1109/access.2023.3307577

**Published:** 2023-08-22

**Authors:** JIAHAO LIN, QI MIAO, CHUTHAPORN SURAWECH, STEVEN S. RAMAN, KAI ZHAO, HOLDEN H. WU, KYUNGHYUN SUNG

**Affiliations:** 1 Department of Radiological Sciences, University of California at Los Angeles, Los Angeles, CA 90095, USA; 2 Department of Electrical and Computer Engineering, University of California at Los Angeles, Los Angeles, CA 90095, USA; 3 Department of Radiology, The First Affiliated Hospital of China Medical University, Shenyang, Liaoning 110001, China; 4 Department of Radiology, Faculty of Medicine, Chulalongkorn University, Bangkok 10330, Thailand; 5 Division of Diagnostic Radiology, Department of Radiology, King Chulalongkorn Memorial Hospital, Bangkok 10330, Thailand

**Keywords:** Generative adversarial networks, magnetic resonance imaging, turbo spin echo, slice profile, super-resolution

## Abstract

High-resolution magnetic resonance imaging (MRI) sequences, such as 3D turbo or fast spin-echo (TSE/FSE) imaging, are clinically desirable but suffer from long scanning time-related blurring when reformatted into preferred orientations. Instead, multi-slice two-dimensional (2D) TSE imaging is commonly used because of its high in-plane resolution but is limited clinically by poor through-plane resolution due to elongated voxels and the inability to generate multi-planar reformations due to staircase artifacts. Therefore, multiple 2D TSE scans are acquired in various orthogonal imaging planes, increasing the overall MRI scan time. In this study, we propose a novel slice-profile transformation super-resolution (SPTSR) framework with deep generative learning for through-plane super-resolution (SR) of multi-slice 2D TSE imaging. The deep generative networks were trained by synthesized low-resolution training input via slice-profile downsampling (SP-DS), and the trained networks inferred on the slice profile convolved (SP-conv) testing input for 5.5x through-plane SR. The network output was further slice-profile deconvolved (SP-deconv) to achieve an isotropic super-resolution. Compared to SMORE SR method and the networks trained by conventional downsampling, our SPTSR framework demonstrated the best overall image quality from 50 testing cases, evaluated by two abdominal radiologists. The quantitative analysis cross-validated the expert reader study results. 3D simulation experiments confirmed the quantitative improvement of the proposed SPTSR and the effectiveness of the SP-deconv step, compared to 3D ground-truths. Ablation studies were conducted on the individual contributions of SP-DS and SP-conv, networks structure, training dataset size, and different slice profiles.

## I. INTRODUCTION

Spin-echo-based acquisitions, such as turbo spin-echo (TSE) or fast spin-echo (FSE) imaging, are preferred for clinical magnetic resonance imaging (MRI) image interpretation for high spatial and contrast resolution for the detection of pathology [1] Three-dimensional (3D) TSE imaging is limited by its long imaging time and related blur image artifact associated with patient motion [2], [3], [4] Instead, multi-slice two-dimensional (2D) TSE imaging is the standard for a range of clinical applications due to its spin-echo-based acquisitions with high contrast and high in-plane resolution (e.g., 0.3–1 mm). However, a stack of 2D slices in a multi-slice 2D acquisition typically has a thicker through-plane resolution (e.g., 3–6 mm), yielding low-resolution (LR) multi-planar reformation (MPR) with staircase artifact due to elongated voxels [4], [5]. As a result, multiple stacks of 2D TSE scans are often acquired in multiple orthogonal imaging planes (e.g., axial, coronal, and sagittal planes), and in some applications, up to five imaging planes (axial, coronal, sagittal and two oblique planes) [5], [6], [7]. These approaches increase the overall scan time, decrease patient comfort, and can also limit the streamlined interpretation of images (e.g., radiologists may need to draw a region of interest (ROI) separately on multiple 2D scans from different orientations). Therefore, methods that achieve super-resolution (SR) transformation of a single multi-slice 2D TSE scan into a high-resolution (HR) isotropic 3D MRI will be valuable to reduce overall imaging time and improve the interpretation of TSE-based MRI images.

Super-resolution reconstruction (SRR) with slice-to-volume registration (SVR) methods have been established to reconstruct a single 3D SR volume from multiple 2D MRI scans [7], [8], [9], [10], [11] Slice-profile downsampling has been proposed to transform SR volume back to LR 2D volumes for fidelity constraints. These SVR algorithms are iterative and require multiple 2D MRI scans, thus increasing the acquisition time and reconstruction time. In contrast, deep-learning-based super-resolution (SR) showed promises in SR of 3D MRI or in-plane 2D MRI [12], [13], [14], [15], [16], [17], [18], [19], [20], [21], [22], [23], [24]. However, applying them to through-plane 2D MRI is non-trivial because of the imperfect slice-selection profile [25].

In this work, we propose a novel slice-profile transformation super-resolution (SPTSR) framework. The SPTSR framework enables the application of deep learning super-resolution to a single stack of multi-slice 2D TSE MRI to achieve 3D isotropic super-resolution by using training inputs synthesized by a realistic representation of the low-resolution through-plane images and slice-profile-transformation based inference pipeline. As multiple orthogonal imaging planes are commonly used in clinical multi-slice 2D TSE MRI, we apply slice-profile-transformation based downsampling (SP-DS) to multi-slice 2D coronal TSE scans as training input to the deep generative model and test our proposed generative network on multi-slice 2D axial TSE scans, reformatted to the coronal plane. We use multi-slice 2D T2-weighted (T2w) prostate MRI, and our aim is to achieve super-resolution 3D imaging with an isotropic resolution of (0.625mm)^3^ from a single multi-slice 2D T2w MRI scan of 3.6mm slice spacing. In addition, we simulated 2D T2w MRI with a large dataset of 3D T2w MRI scans to quantitatively evaluate SPTSR with ground-truth images.

The main contributions of our SPTSR framework include that

1. We used a dedicated observation model (i.e., an appropriate definition of a 2D excitation profile) that enables coronal (or axial) scans for supervised training via SP-downsampling to perform SR of axial (or coronal) scans.
2. Both the training and inference images are blurred via SP-downsampling and SP-conv to match the slice profile kernel in the two orthogonal directions, and the output images are then deblurred via SP-deconv to achieve super-resolved isotropic 3D imaging.
3. The purpose and necessity of SP-downsampling, SP-conv, and SP-deconv, collectively referred to as SPTSR, are proved both in theory and experimental results (Figs. 6 and 9).
4. With extensive visual, qualitative, and quantitative comparisons, we establish that SPTSR significantly improves the quality of SR images when compared to the SMORE method [26] and k-space zero-fill (KS-ZF) trained networks.
5. The feasibility of using only one multi-slice 2D TSE scan for a high-resolution MPR is shown using the SPTSR framework. This can potentially save total MRI scan time considerably as it negates the necessity of scanning multiple stacks of 2D TSE scans in orthogonal imaging planes.

## II. RELATED WORKS

Existing super-resolution reconstruction (SSR) methods generally require multiple 2D MRI scans to iteratively register and reconstruct a single 3D SR volume. Greenspan et al. proposed an inter-slice super-resolution algorithm that utilized three stacks of multi-slice 2D images, each volume shifted by a sub-pixel amount in the slice direction [27]. An iterative back-projection algorithm was used to reconstruct the high-resolution image volume [27]. Rousseau et al. and Jiang et al. developed slice-to-volume registration (SVR) to register multiple sets of scans from three orientations onto a single high-resolution 3D volume [8], [9]. Gholipour et al. used a total of scans from three orientations to perform SVR and iterative SSR [10], [11]. They proposed a slice acquisition model, including SP-downsampling, which was used to iteratively transform the SR volume back to LR input volume, and enforce the fidelity constraints [10], [11]. For lengthy cardiac cine scans, motion-compensated reconstruction was proposed to combine multiple 2D cine scans into a 3D cine volume [28]. Automated pipelines with CNN-based initial SVR estimation and CNN-based localization and segmentations were also developed [7], [29], but iterative SSR was still performed. SSR frameworks demonstrated qualitative and quantitative improvement from the 2D scans [30], [31]. However, these SSR approaches used multiple 2D scans and iterative SSR algorithms, which significantly increased the acquisition time and the reconstruction time.

Deep learning SR algorithms are the state-of-the-art for SR in natural images and have become increasingly popular for SR in MRI [19], [32]. Many studies focused on SR of 3D MRI or in-plane SR for 2D MRI and showed promise in achieving high in-plane resolution with single-image SR [12], [13], [14], [15], [16], [17], [18], [19], [20], [21], [22], [23], [24]. To synthesize the LR training input from the HR reference images, these works used either averaging-based or interpolation-based downsampling [12], [13], [14], [15], [16], [17], [18] or KS-ZF downsampling [19], [20], [21], [22], [23], [24]. However, applying them to achieve high through-plane resolution is challenging with multi-slice 2D TSE imaging datasets because super-resolution algorithms were trained and tested along with the frequency and phase encoding directions. Frequency and phase encoding schemes divide the voxels evenly in the frequency domain, where they are continuous, uniform, and non-overlapping [33]. In this case, training input for super-resolution can be easily synthesized by downsampling HR reference images. In contrast, multi-slice 2D TSE imaging is realized by applying a radiofrequency (RF)-excitation pulse with a slice-selection profile for each individual slice [25]. Due to MR hardware limitations, the slice-selection profiles may not be sharp-edged and can overlap with adjacent slices [25]. To compensate, slice spacing greater than the slice thickness is often used to avoid the slice overlapping, resulting in physical discrepancies between the evenly-spaced super-resolution and through-plane resolution of multi-slice 2D TSE imaging. Training input for super-resolution cannot be easily synthesized by simple downsampling due to the fundamental difference between actual through-plane resolution and synthesized low-resolution images.

Several studies have proposed to increase the through-plane resolution of multi-slice 2D MRI with learning-based super-resolution. Jurek et al. developed a CNN-based super-resolution reconstruction using thick slices [34], and Zhang et al. developed a GAN-based super-resolution algorithm with multiple 2D scans [35]. Bhatia et al. proposed SR of cardiac MRI with coupled dictionary learning [36]. These three methods all generate the LR simulated input with averaging-based or interpolation-based downsampling. While they show impressive with simulated testing data, they did not perform testing on actual 2D scans. Sood and Rusu [5], [37] used training from conventional k-space zero-filled downsampled HR axial prostate MRI to test on reformatted multi-slice 2D coronal MRI. Although excellent validation results were shown, the testing results did not fully resolve the staircase artifact and were visibly much noisier compared to the reference images. SMORE is the state-of-the-art SR algorithm that super-resolves one stack of 2D slices to an isotropic 3D volume [26]. SMORE trained on downsampled HR axial slices and infer on the reformatted 2D coronal slices. It attempted to factor in staircase artifacts by applying a self-supervised anti-aliasing step (SAA) but did not fully consider slice profiles during the downsampling and inference. Thus, although it demonstrated good results in brain MRI, certain anatomically challenging MRI scans may not be applicable due to the limitations of the training data. Our proposed SPTSR will be extensively compared with SMORE as the state-of-the-art.

## III. METHODS

**FIGURE 1.**
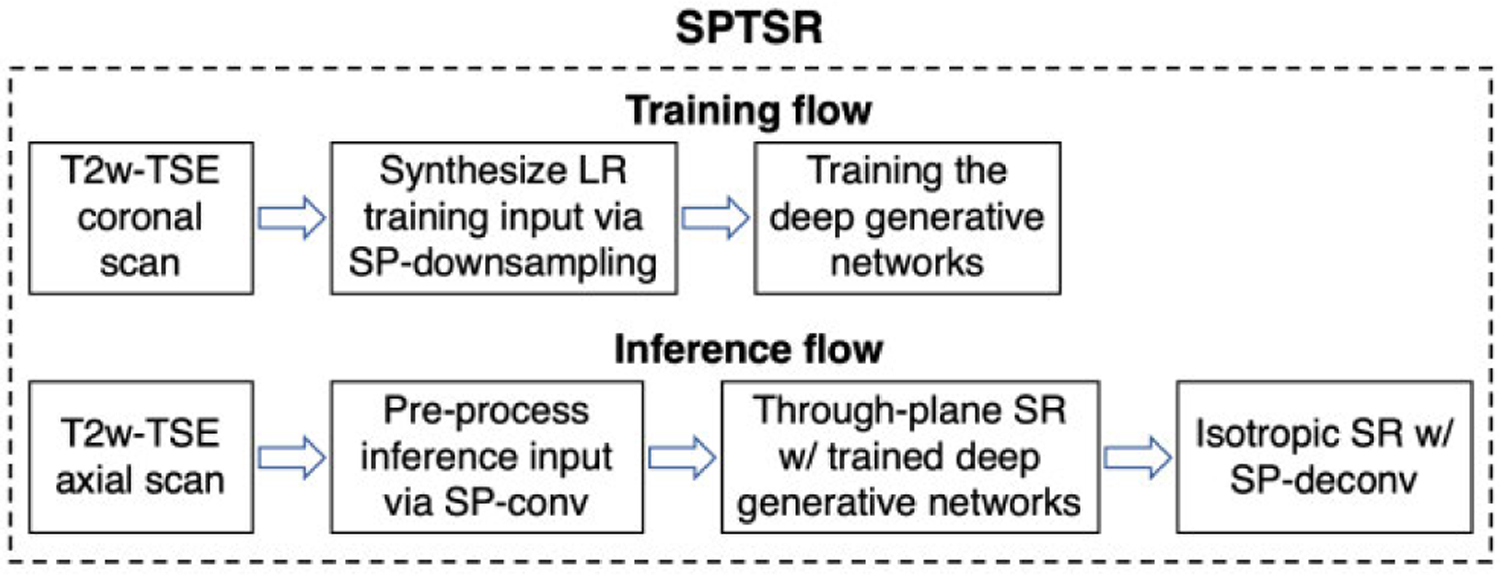
The overall SPTSR framework, with training flow (top) and inference flow (bottom).

We describe the complete framework of our proposed SPTSR in detail, summarized in Fig. 1. Current clinical MRI scans, including prostate, brain, and placenta MRI, commonly include several stacks of multi-slice 2D TSE MRI scans with two or three orthogonal orientations (e.g., axial, coronal, and sagittal) to compensate for low through-plane resolution. The goal of the proposed SPTSR framework is to train from one orientation of a single stack of 2D slices (e.g., a coronal MRI scan) and use the orthogonal orientation (e.g., an axial MRI scan) to infer an isotropic high-resolution 3D imaging volume. The isotropic 3D imaging volume can be transformed into other orientations via MPR. For this to work:

- We designed a novel slice-profile transformation super-resolution (SPTSR) framework, with a pre-processing SP-downsampling for an orthogonal stack of multi-slice 2D scan as the training input, a pre-processing SP-conv for the inference input, and a post-processing SP-deconv for the inference output.
- We utilized a large clinical prostate MRI dataset, consisting of axial and coronal stacks of multi-slice 2D TSE MRI scans, for training, validation, and testing.
- We designed the WGAN-GP scheme for the training of our deep generative networks.

### A. SLICE-PROFILE TRANSFORMATION

We define a stack of multi-slice 2D coronal images as *I*_*cor*_ (with high resolution in the SI-direction) and a stack of multi-slice 2D axial images as *I*_*ax*_ (with low resolution in the SI-direction). A set of *I*_*cor*_ is used for training and validation, and *I*_*ax*→*cor*_, reformatted from axial to coronal planes is used for the input to the inference model. Although not shown, the principle would hold the same way for other orientations (e.g., *I*_*ax*→*sag*_, *I*_*cor*→*ax*_, etc.). We define *V* as the underlying isotropic high-resolution 3D imaging volume with a matrix size of *N*_*LR*_ × *N*_*AP*_ × *N*_*SI*_ and field-of-view (FOV) of *F*_*LR*_ × *F*_*AP*_ × *F*_*SI*_. Then, *I*_*cor*_ and *I*_*ax*_ at the pixel/slice indices, *x*, *y*, *z*, with the same FOV are expressed as:

(1) $${I_{\text{cor}}(x,y,z)|}_{x\in \left[1,N_{LR}\right],y\in \left[1,NS_{AP}\right],z\in \left[1,N_{SI}\right]}\;=\sum_{j=y\times DS_{AP}-\left\lfloor \frac{L}{2}-1\right\rfloor }^{j=y\times DS_{AP}+\left\lfloor \frac{L}{2}\right\rfloor }PSF_{AP}(j)V(x,j,z),$$

(2) $${I_{ax}(x,y,z)|}_{x\in \left[1,N_{LR}\right],y\in \left[1,N_{AP}\right],z\in \left[1,NS_{SI}\right]}\;=\sum_{k=z\times DS_{SI}-\left\lfloor \frac{L}{2}-1\right\rfloor }^{k=z\times DS_{SI}+\left\lfloor \frac{L}{2}\right\rfloor }PSF_{SI}(k)V(x,y,k),$$

where *PSF*_∗_ is the normalized one-dimensional (1D) slice profile for a given RF-excitation pulse, *NS*_∗_ is the number of slices, and *DS*_∗_ is the spacing between slices in coronal (* = AP) and axial (* = SI) scans. *L* is the slice thickness, full-width-half-max (FWHM) of *PSF*. *DS*_*x*_ becomes same as L if there is no slice gap. *j, k* are upsampled indices to account for non-integer indices after scaling. The *PSF* is approximated as truncated sinc function. While the true slice profile is possible to compute by the combination of slice profiles of RF excitation and refocusing pulses, the difference between exact and approximated ones would be subtle and beyond the scope of our work as we apply projection to all signals.

**FIGURE 2.**
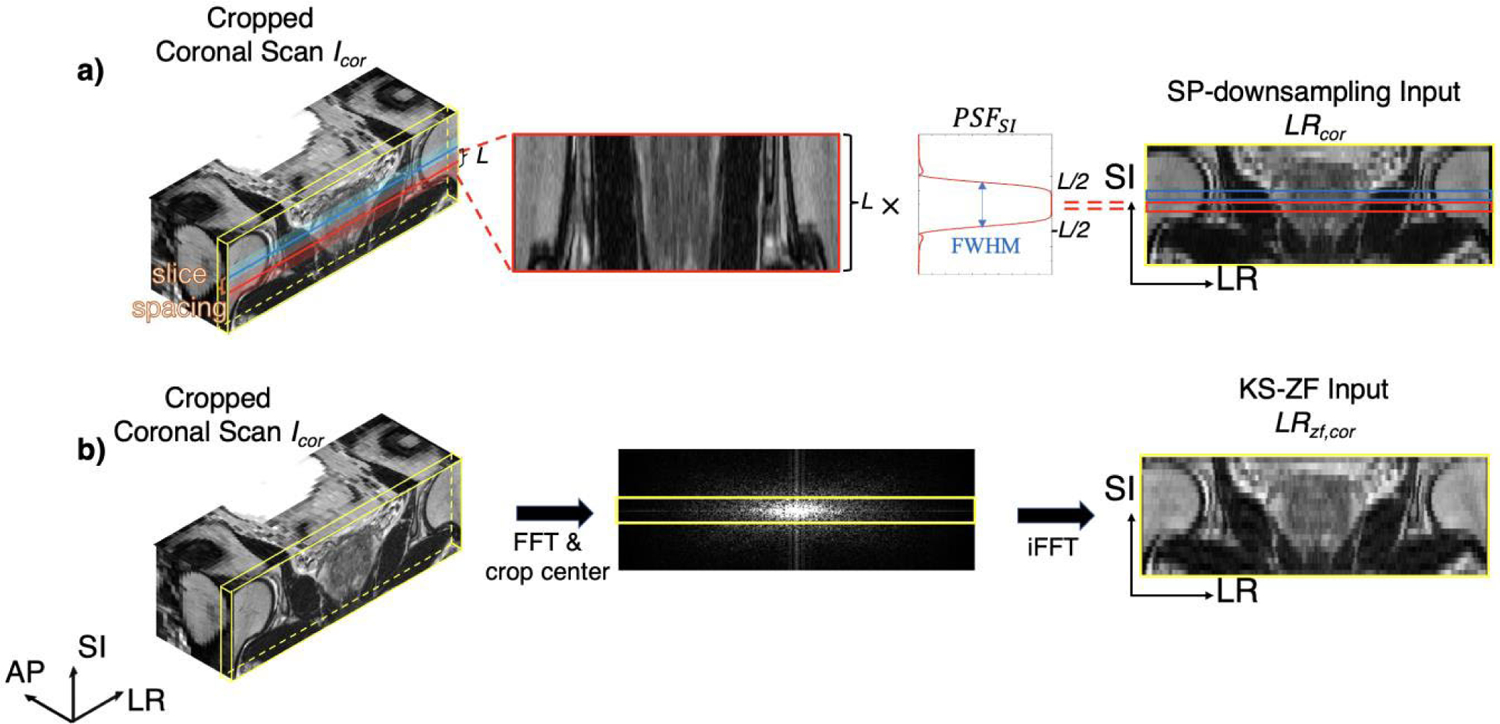
a) The proposed SP-downsampling method. Each line of pixels is acquired by multiplying the slice profile *PSF*_*SI*_ of length *L*, to the same physical location on the cropped coronal scan; b) The KS-ZF downsampling method. Each slice of image is transformed to the frequency domain via FFT, cropped its center lines and iFFT back to the downsampled image.

**FIGURE 3.**
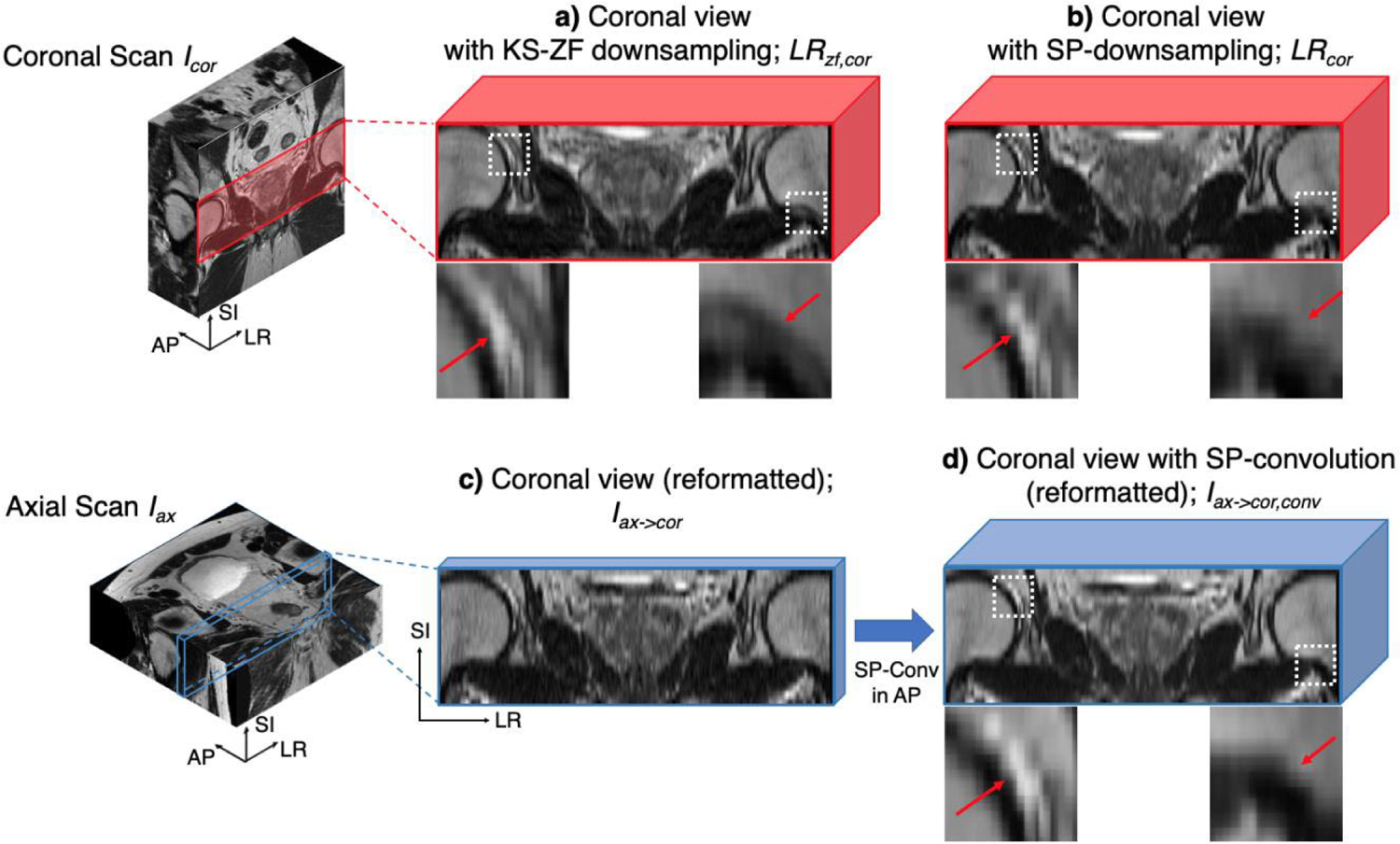
The down-sampling method visual comparison. a) the conventional KS-ZF downsampled image patch, b) the SP-downsampled image patch, c) the reformatted axial patch, and d) the SP-convolved axial patch. Thickness in each patch represents the voxel thickness in the AP-direction. All patches are bilinear interpolated to demonstrate the visual differences.

Previous studies attempted to synthesize *I*_*ax*→*cor*_ by down-sampling *I*_*cor*_ via k-space zero-filling (KS-ZF) [21], [22], [23], [24]. The KS-ZF transforms the source image to the frequency domain via FFT, crops the center at 1/up-sampling factor, and converts it back to the image domain via iFFT, as illustrated in Fig. 2b. Essentially, the KS-ZF in the axial plane *LR*_*zf*_,_*cor*_ can be expressed as applying a 1D-lowpass filter with a rectangular window to *I*_*cor*_ in the SI-direction:

(3) $$LR_{zf,\text{cor}}=LP_{SI}\left(I_{\text{cor}}\right),$$

where *LP*_*SI*_ is the 1D low-pass filtering along the SI-direction. *LR*_*zf,cor*_ and *I*_*cor*_ form the conventional LR-HR training pair for KS-ZF trained deep learning networks. However, *LR*_*zf*,*cor*_ is inherently different from its reformatted version from the axial scan *I*_*ax*→*cor*_, as shown in Fig. 3a and 3c. In particular, *I*_*ax*→*cor*_ contains weaving patterns (Fig. 3c) while *LR*_*zf,cor*_ is smooth overall (Fig. 3a; see the red arrows). This is because:

- *LR*_*zf,cor*_ does not account for the imaging characteristics due to the convolution of *PSF*_*SI*_ on *I*_*ax*_,
- the 3 mm slice thickness of *LR*_*zf*_,_*cor*_ (FWHM of *PSF*_*AP*_) in the AP-direction is five times thicker than the 0.6 mm slice thickness of *I*_*ax*→*cor*_ in the AP-direction, and
- the 3 mm voxel spacing of *LR*_*zf,cor*_ (FWHM of *PSF*_*SI*_) in the SI-direction is different than the 3.6 mm voxel spacing of *I*_*ax*→*cor*_ in the SI-direction (*DS*_*SI*_).

**FIGURE 4.**
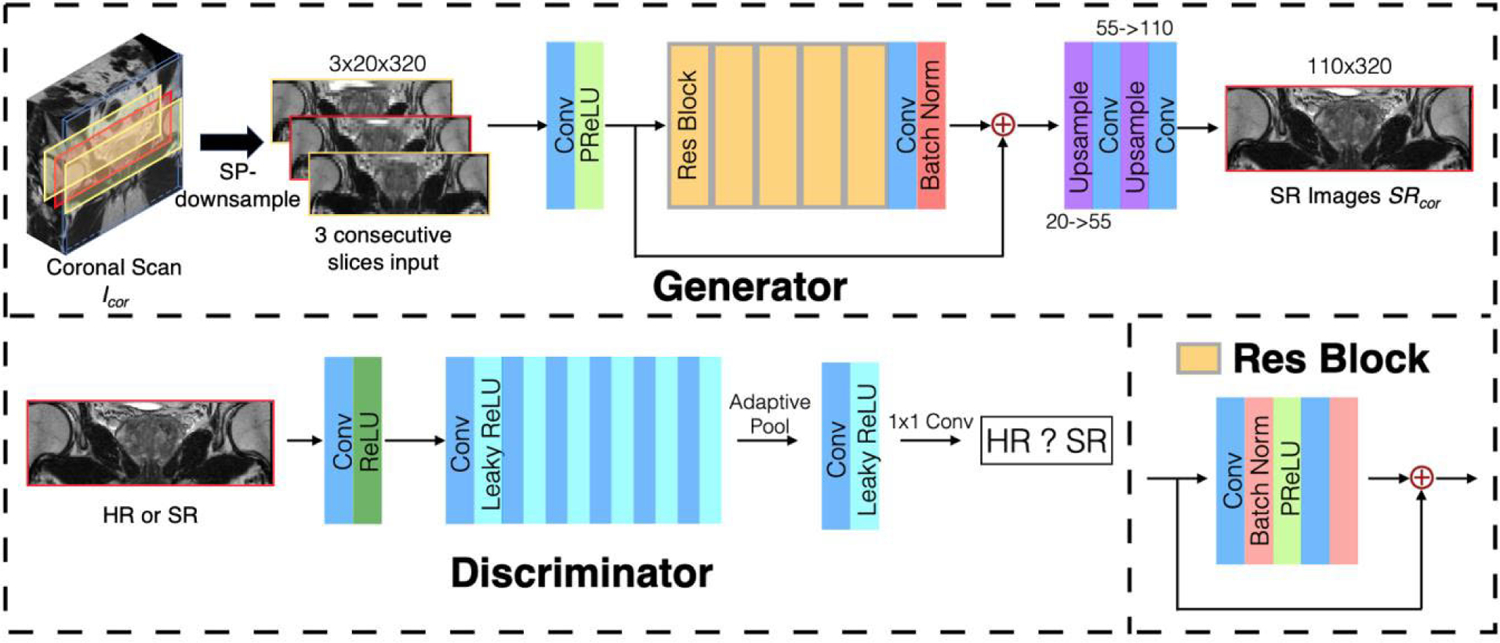
The WGAN-GP based deep generative networks architecture of our proposed SPTSR.

To address the above three differences, we transform both *I*_*cor*_ and *I*_*ax*_ to the common LR image domain by considering both *PSF*_*AP*_ and *PSF*_*SI*_. To synthesize the training input with *I*_*cor*_, we convolve *I*_*cor*_ with *PSF*_*SI*_ (Fig. 2a):

(4) $${{\text{LR}}_{\text{cor}}(x,y,z)|}_{x\in \left[1,N_{LR}\right],y\in \left[1,NS_{AP}\right],z\in \left[1,NS_{SI}\right]}\;=\sum_{k=z\times DS_{SI}-\left\lfloor \frac{L}{2}-1\right\rfloor }^{k=z\times DS_{SI}+\left\lfloor \frac{L}{2}\right\rfloor }PSF_{SI}(k)I_{\text{cor}}(x,y,k),$$

where *LR*_*cor*_ and *I*_*cor*_ form a LR-HR training pair for our deep generative networks (Fig. 4). The SR training output (*SR*_*cor*_) has the same dimension and voxel size as the HR reference (*I*_*cor*_).

**FIGURE 5.**
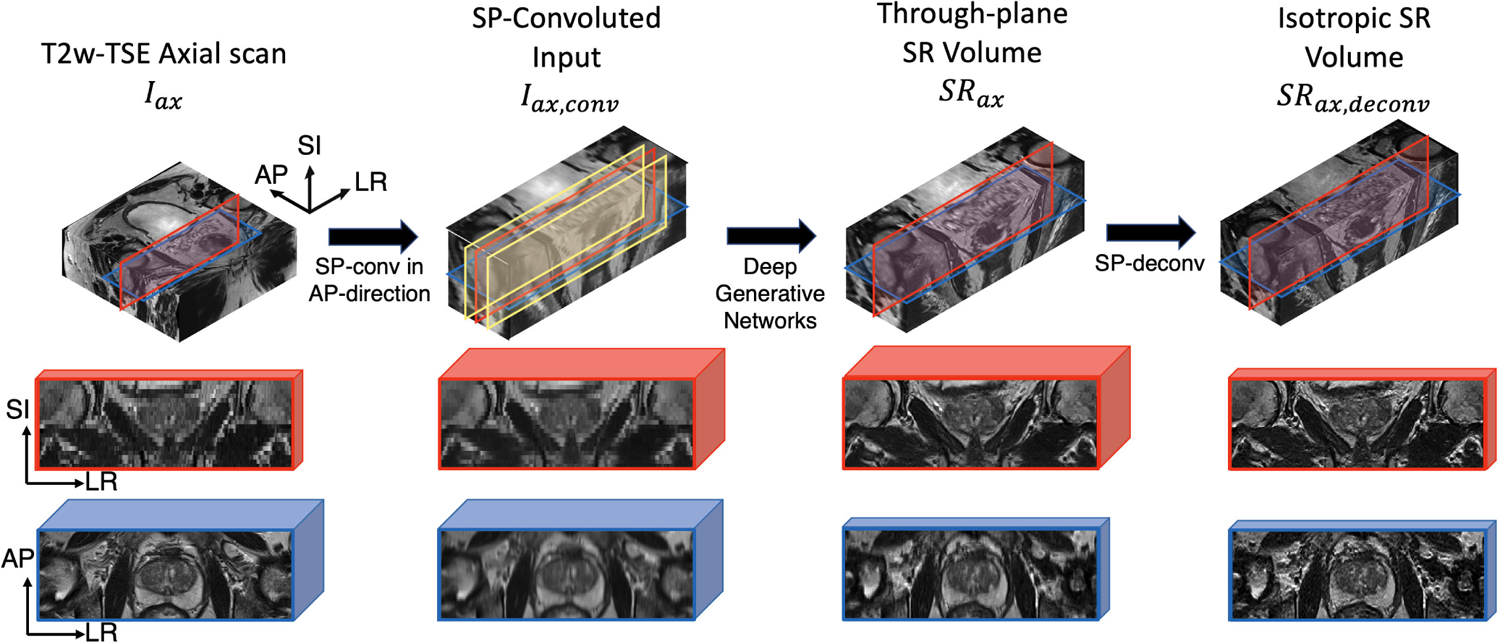
The proposed SR inference flow. T2w-TSE axial scan is cropped and SP-convolved in the AP-direction to prepare the input for the deep generative networks. The output of the networks is still convolved in the AP-direction and is then transformed to isotropic SR volume via SP-deconvolution. Red patches represent coronal views and blue patches represent axial views. Patch thickness represents the voxel thickness in the through-plane of the patch.

For inference, we convolve *I*_*ax*_ with *PSF*_*AP*_ to form the convolved input *I*_*ax,conv*_ (Fig. 5), defined as:

(5) $${I_{ax,\text{conv}}(x,y,z)|}_{x\in \left[1,N_{LR}\right],y\in \left[1,N_{AP}\right],z\in \left[1,NS_{SI}\right]}\;=\sum_{j=y\times DS_{AP}-\left\lfloor \frac{L}{2}-1\right\rfloor }^{j=y\times DS_{AP}+\left\lfloor \frac{L}{2}\right\rfloor }PSF_{AP}(j)I_{ax}(x,j,z).$$

Note that the dimension is *NY* in the AP-direction, and we keep the matrix size of *I*_*ax*_ by applying a sliding window for the convolved input *I*_*ax,conv*_.

Comparing the training input *LR*_*cor*_ and the inference input *I*_*ax,conv*_ (Fig. 3b and 3d), both contain similar weaving artifacts in the SI-direction by considering both *PSF*_*AP*_ and *PSF*_*SI*_. This is because the three physical problems are addressed by:

- both *LR*_*cor*_ and *I*_*ax,conv*_ that have multiplied with *PSF*_*SI*_ and *PSF*_*AP*_,
- both *LR*_*cor*_ and *I*_*ax,conv*_ that have the same 3 mm slice thickness in the AP-direction of FWHM of *PSF*_*AP*_, and
- both *LR*_*cor*_ and *I*_*ax,conv*_ that have the same 3.6 mm voxel spacing in the SI-direction of *DS*_*SI*._

Thus, we ensure the input to the networks in training/validation, and the input in the inference flow are of the consistent intrinsic voxel size and spacing.

### B. DEEP GENERATIVE NETWORKS

Our SPTSR framework is model-agnostic, and the specific network architecture for deep generative networks was not the focus of our study. We adopted deep generative networks architecture largely from SRGAN [38], as shown in Fig. 4, with the three key differences. First, we used three consecutive low-resolution images as input, with the middle slice being the targeted input. By adding the adjacent slices to the original input, the networks can learn the spatial relationship between image slices. Because of imperfect slice excitation, the voxel information was intertwined between adjacent slices, further helping the model to generate super-resolution images. Compared to feeding the whole image volume into the networks, 3-slice input significantly lowers the overall graphical memory usage. Secondly, batch normalizations and the last sigmoid activation function were removed from the discriminator network because our networks were trained using WGAN-GP [39]. Lastly, the upsampling blocks in the generator model were modified to 1D anisotropic upsampling.

### C. INFERENCE FLOW

The overall inference flow is shown in Fig. 5. Similar to the training 3-slice input, the input for inference contains three SP-convolved slices, including a center slice (red) and two adjacent slices (yellow). Note that the slices between training/validation input *LR*_*cor*_ have slice spacing of *F*_*AP*_*/NS*_*AP*_, so for the inference input *I*_*ax,conv*_, the adjacent slices are also convolved at the physical distance of *F*_*AP*_*/NS*_*AP*_ from the middle slice. Because the LR-HR training pairs are *LR*_*cor*_ and *I*_*cor*_, the output from deep generative networks has the same voxel dimension and characteristics of *I*_*cor*_. This applies to both the training/validation and inference pipeline. Each coronal plane slice of the inference output *SR*_*ax*_ is at the same resolution, matrix size and contrast compared to the cropped coronal scan *I*_*cor*_. In addition, the inference output *SR*_*ax*_ is convolved in the AP-direction with an elongated voxel size, with the same matrix size *F*_*AP*_*/N*_*AP*_ of *I*_*ax*_ in AP-direction. Thus, the inference through-plane SR output *SR*_*ax*_ not only synthesizes the coronal scan, but has an isotropic voxel spacing. To fully utilize this isotropic voxel spacing characteristic, deconvolution, with the 1D slice profile *PSF*_*AP*_ (Fig. 5). The end product is the isotropic SR volume *SR*_*ax,deconv*_. By using this iterative noise-robust Richardson-Lucy deconvolution method [40], [41], we transform the *SR*_*ax*_ with an elongated voxel size of $\left(\frac{F_{LR}}{N_{LR}},\frac{F_{AP}}{NS_{AP}},\frac{F_{SI}}{N_{SI}}\right)$, to an isotropic high-resolution image volume *SR*_*ax,deconv*_, with isotropic voxel size $\left(\frac{F_{LR}}{N_{LR}},\frac{F_{AP}}{N_{AP}},\frac{F_{SI}}{N_{SI}}\right)$.

### D. MULTI-SLICE 2D EXPERIMENTS

#### 1) MRI DATASET

We retrospectively reviewed clinical prostate MRI scans from March 2013 to December 2018 at a single academic institution and identified a total of 3,895 clinical subjects with 4,878 paired stacks of axial and coronal images using the multi-slice 2D T2-weighted turbo spin-echo (T2w-TSE) sequence. Institutional Review Board (IRB) approval was obtained for the study. The MRI scans were performed on one of several Siemens 3 Tesla scanners (including Prisma, Skyra, Vida, and Trio; Siemens Healthineers, Erlangen, Germany). Most clinical subjects had one pair of coronal and axial scans, and some subjects had more than one pair of scans at different time points. Both axial and coronal scans were based on the same imaging sequence except for the imaging-plane orientations. Each stack included 20 slices of T2w-TSE images, with an in-plane resolution of 0.625 × 0.625 *mm*^2^ (the matrix size of 320 × 320). The slice thickness was 3 mm with a slice spacing of 3.6 mm (i.e., a nominal gap of 0.6 mm between adjacent slice boundaries). No parallel imaging was used for reconstruction. The MRI sequence parameters are shown in Table 1.

#### 2) DATA PREPARATION

The training/validation/testing splits were 3,453/392/50 from 3,895 clinical subjects. Training and validation used coronal scans. All of the training and validation coronal scans were cropped (320 to 110) in the z-direction to the same physical coverage distance as the axial scans. They were then downsampled by both the conventional KS-ZF and SP-downsampling methods to create separate training/validation datasets. Each coronal scan had 20 slices, generating 18 three-slice input samples.

The testing dataset used axial scans, and scans with strong inter-slice motion artifacts were manually excluded. The axial scans were reformatted to coronal orientation, which produced 320 slices of 20 × 320 reformatted images. They are also cropped (320 to 110) to have only imaging volumes of prostates overlapping with the coronal scans. The high-resolution coronal images were used as visual references.

#### 3) TRAINING SCHEME

The deep generative networks were trained using the WGAN-GP scheme [39]. Thus, the discriminator loss included a weighted sum of the adversarial and gradient penalty loss. The generator loss included the weighted sum of the adversarial loss, mean-square-error (MSE) loss, and the VGG perceptual loss, where the weights were [10e-3, 1, 10e-6] respectively. The VGG perceptual loss showed excellent performance as a perceptual loss for super-resolution tasks [38], [42]. MSE of VGG-23 network output was used as the perceptual loss. The network was trained with 100 epochs, and the actual epoch was determined with the lowest validation MSE loss. The batch size was set to 64. Adam optimizer was used. The learning rate was 10e-4.

#### 4) EXPERT READER STUDY

We designed our expert reader study, similar to the recent studies [3], [43], to assess the through-plane SR results with scans reformatted to coronal views. After a few training sessions, two genitourinary radiologists (M.Q. and C.S.; each had interpreted 500–1,500 prostate MRI scans with 5+ years of experience) independently assessed four methods: bilinear interpolation (BI), SMORE [26], KS-ZF trained networks (baseline), SPTSR (proposed). Any information indicating the type of processing was removed from all images and randomly shuffled for each subject when they were compared against the visual reference of HR in-plane T2w-TSE coronal scan of the same subject. All five image sets were simultaneously loaded into OsiriX (Pixmeo SARL, Bernex, Switzerland) and the reader scrolled through the coronal slices. Four methods all have a 5.5x number of slices as the original coronal scan, within the same physical distance. In total, 50 subjects (each with one axial scan) of the testing dataset were examined. Diagnostic quality metrics of sharpness (1: severe blurring, 2: moderate blurring, 3: mild blurring, and 4: no blurring), artifacts (1: severe artifacts, 2: moderate artifacts, 3: mild artifacts, and 4: no artifacts), noise level (1: severe noise, 2: moderate noise, 3: mild noise, and 4: no noise) and overall diagnostic image quality (1: severe, 2: moderate, 3: good, and 4: excellent) of each method were scored on a 4-point quality scale. The reader also blindly ranked the overall quality of the four methods against each other. The visual reference of the HR coronal scans was considered as the score 4 in all metrics. The coronal scans were not considered as ground-truth because they were separate scans from the axial scans and did not align precisely in space due to the patient and rectal motion between scans.

Averaged ratings and rankings from two readers were compared between three methods. Mann-Whitney U tests were used to assess the significant differences (p<0.01) between the four methods. Cohen’s Kappa was calculated for inter-reader variability [44].

#### 5) QUANTITATIVE ANALYSIS

Quantitative analysis of the same four methods: BI, SMORE, baseline, and proposed method, was also performed to cross-validate with the qualitative reader study using the same 50-subject testing dataset. Because the visual reference coronal scans were not aligned pixel-by-pixel with the SR images, metrics such as peak signal-to-noise ratio (PSNR) or structural similarity index (SSIM) were not suitable here. Fréchet inception distance (FID) is one of the most common metrics for assessing the quality of images generated by generative models, for both natural images and MRI images [45], [46], [47], [48]. Real and generated images were fed through a pre-trained inception network, and the FID measured the distance of the distributions between their activation output without requiring for pixel-wise alignment with reference [45], [46].

For each method, 1,995 pairs of real and generated images are used to calculate FID. Each pair of images are cropped to 110×320 at the same scanner physical locations. The Mann-Whitney U test was used to assess the statistical differences between the four methods.

### E. 3D SIMULATION EXPERIMENTS

3D simulation experiments were also conducted to further evaluate the effectiveness of SPTSR compared with 3D T2w as ground-truth.

#### 1) MRI DATASET

We retrospectively reviewed clinical prostate MRI scans from March 2013 to December 2018 at a single academic institution and identified a total of 4,637 clinical subjects with 5,848 scans using the 3D T2w-TSE (SPACE) sequence. Institutional Review Board (IRB) approval was obtained for the study. The MRI scans were performed on one of several 3 Tesla scanners (including Prisma, Skyra, Vida, and Trio; Siemens Healthineers, Erlangen, Germany). The sequence parameters are shown in Table 2.

#### 2) DATA PREPARATION

The training/validation/testing splits were 4,080/464/93 from 4,637 clinical subjects. Each 3D scan volume was first interpolated to isotropic grid with voxel resolution of 1mm^3^, with a matrix size of (170 × 170 × 90).

The isotropic 3D volume was then center-cropped and SP-downsampled, with a 3mm truncated sinc PSF and 4mm slice thickness (4× downsampling). The training/validation datasets were SP-downsampled along the SI direction, simulating 2D T2w-TSE axial scans, whereas the testing datasets were SP-downsampled along the LR direction, simulating 2D T2w-TSE sagittal scans.

#### 3) TRAINING AND INFERENCE

Training schemes and inference flows for 3D simulation experiments followed the procedures of 2D T2w-TSE experiments.

The simulated 2D T2w-TSE axial scans for training/validation were SP-downsampled along the LR direction to train/validate the deep generative networks. For testing, the simulated 2D T2w-TSE sagittal scans were inferenced through the trained deep generative networks and SP-deconvolved to reach 4× isotropic super-resolution of the original 1mm^3^ voxel size.

#### 4) QUANTITATIVE ANALYSIS

The simulation experiments have 3D isotropic high-resolution ground-truth volumes. Peak signal-to-noise ratio (PSNR) and normalized MSE (NMSE) were measured for each super-resolution output volume compared to the 3D T2w ground-truth.

For each method of SMORE, before-deconv, and after-deconv (SPTSR), 118 3D volumes from 93 testing subjects were used to calculate volumetric PSNR and NMSE. Mean and standard deviations were calculated, and paired samples t-test were conducted between each pair of method to evaluate statistical significance [49].

### F. ABLATION STUDIES

#### 1) NETWORK ARCHITECTURES

Our proposed deep generative networks are compared against three other popular deep-learning networks structures, such as U-Net [50], ResUNet [51], and SRGAN [38], to demonstrate the compatibility of the SPTSR framework with other deep learning networks architecture. For each of all four networks, it is trained with SP-downsampled training data for up to 60 epochs. The epoch with the minimal MSE loss in the validation dataset has been chosen as the converged trained networks. Synthetic validation input from the validation datasets is used to assess the network structure in the ablation study. LR SP-downsampled images were generated directly from their high-resolution coronal scans. Both PSNR and structural similarity index measure (SSIM) were used to quantify the quality of the network’s structure ablation because they exactly matched with the ground truth HR coronal scan. The number of parameters of the networks is also compared.

SRGAN is a single-image super-resolution (SISR) network, originally for 2D isotropic super-resolution. The upsampling blocks are replaced with anisotropic upsampling blocks used in our proposed networks to match the input to output image size. Similarly, the anisotropic upsampling blocks are concatenated to the front of the networks for both U-Net and ResUNet because they are designed for the same input and output size. The input images for both U-Net and ResUNet have been replaced with 3-slice input, and both networks also use the same training scheme as the proposed networks for a fair comparison.

#### 2) SIZE OF THE TRAINING DATASET

Acquiring a large dataset for training can be practically challenging [26]. We examined four different sizes of training datasets (N=10, 100, 1,000, and 3,453) to understand how much data is sufficient to train the proposed networks. The same validation dataset of 392 subjects was used to quantitatively assess the effectiveness of the training dataset. The same batch size was used in training, and the validation MSE loss was plotted against the number of training steps.

#### 3) SLICE PROFILES

The truncated sinc slice profile used in this study was simulated via Bloch equations, an approximated version of the actual slice profile [25]. The Gaussian PSF slice profile was also considered a different approximation of the actual slice profile for TSE sequences [11], [27]. Slice thickness and pixel size were used to investigate the impact of different slice profile approximations, and the following ablation study was conducted on different slice profiles.

The proposed networks were trained with training data downsampled with simulated truncated sinc PSF, and the Gaussian PSF. Then, the two trained networks were both validated with validation dataset downsampled with truncated sinc PSF, and the Gaussian PSF. SSIM and PSNR values were calculated for each of the four cases.

## IV. RESULTS

### A. MULTISLICE 2D EXPERIMENTS

#### 1) THROUGH-PLANE SR RESULTS

**FIGURE 6.**
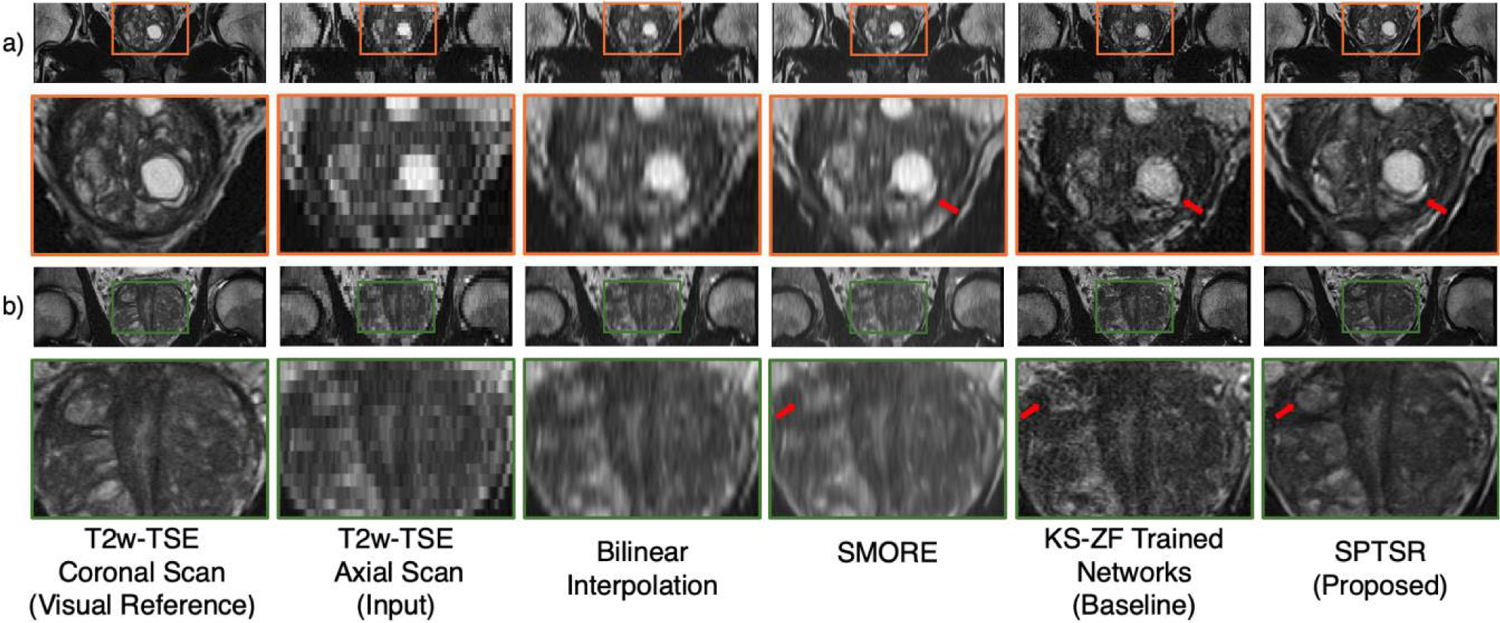
The through-plane SR testing results with reformatted T2w axial scan input. a) and b) represents two image slices from two different testing subjects. From left to right: The T2w coronal scan of the subject, as a visual reference; the testing reformatted T2w axial scan as the inference input; the bilinear interpolation of the input; SMORE [26]; the baseline inference output with KS-ZF trained networks; the proposed SPTSR inference output with SP-downsampling trained network, and SP-convoluted inference input. Red arrows indicate the structural differences between the baseline results and the proposed results.

The output images after 5.5x through-plane SR are shown in Fig. 6. The T2w coronal and reformatted T2w axial scan images are shown as visual references, and the output results from bilinear interpolation, SMORE [26], KS-ZF trained networks, and SPTSR are compared to each other. The T2w coronal scan is used as a visual reference, not a ground truth, as it was acquired in a different scan. Both SMORE and KS-ZF trained networks removed most staircase and smearing artifacts compared to the simple bilinear interpolation but failed to reconstruct small structures within the prostate with amplified noise. The HR images produced by SPTSR are successfully containing small structures within the prostate, visually close to the T2w coronal scan images.

#### 2) EXPERT READER STUDY

**FIGURE 7.**
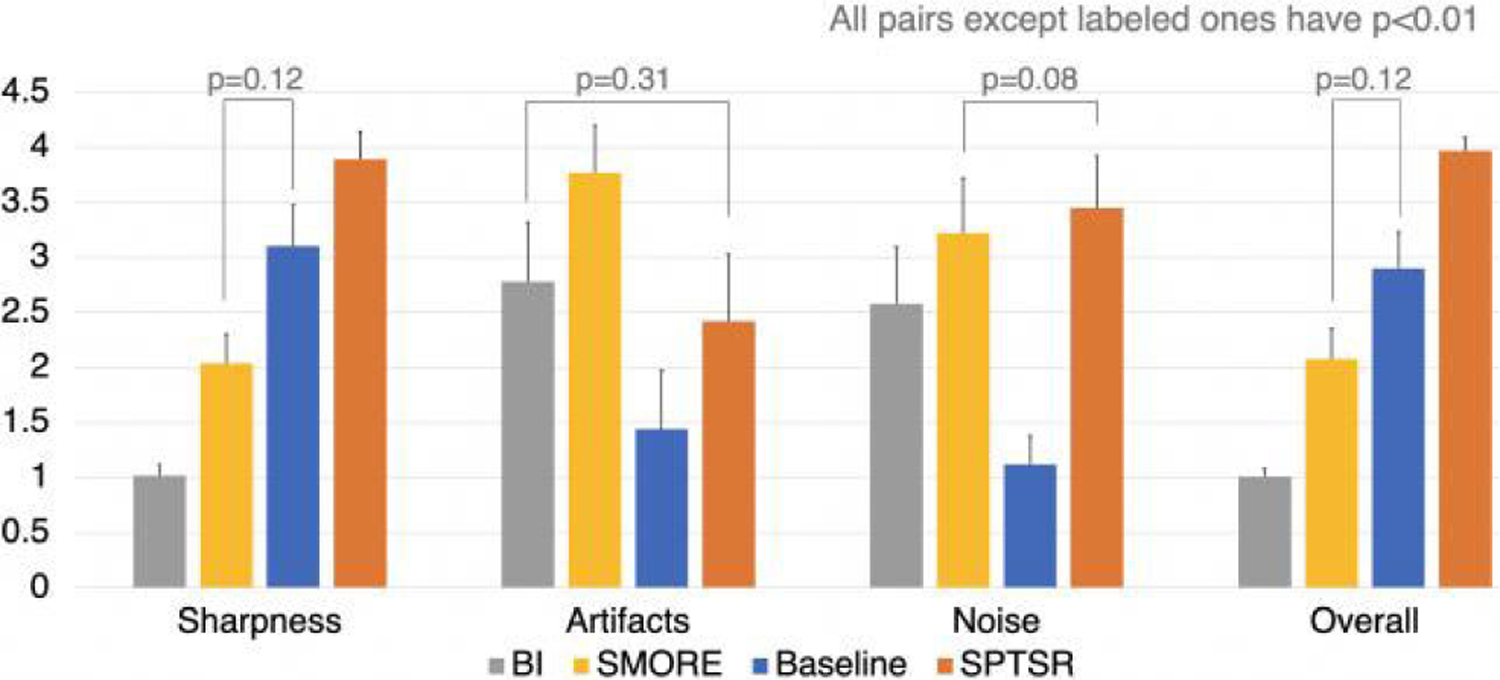
Two radiologists qualitatively assessed the diagnostic quality of Bilinear Interpolation (BI), SMORE [8], KS-ZF trained networks (baseline), SPTSR (proposed), for sharpness, artifacts, noise and overall image quality on a 1 to 4 scale (higher the better). The ratings were averaged from two readers. Error bar represents the standard deviations. Mann-Whitney U tests assessed whether the average scores were significantly different (p<0.01) among the four groups.

The image quality assessment of the output results (1: severe, 2: moderate, 3: good, and 4: excellent) conducted by the expert readers is shown in Fig. 7. In a blinded fashion, the SPTSR method received an almost perfect overall image quality score (all cases received 4.0 except three cases, which received 3.5). The SPTSR method has significantly better overall image quality scores (p<0.01) compared to all other methods (BI, SMORE, and KS-ZF). For ranking results, the SPTSR method ranked the best in 48 cases agreed by both radiologists and best and second-best in 2 cases by two radiologists.

The proposed SPTSR method is significantly better compared to the baseline KS-ZF trained network in terms of sharpness, noise level, and overall image quality (p<0.01). Although SMORE had the best artifact score, its overall image quality was limited by its poor sharpness quality, thus having its overall score worse than the baseline, and significantly worse than the proposed method. The image quality assessment substantially agreed between the two readers with Cohen’s Kappa of 0.72 (95% confidence interval: 0.66–0.78).

#### 3) QUANTITATIVE ANALYSIS

The testing image quantitative assessment with FID is shown in Table 3. For each method, the FIDs of 1,995 images are shown as mean ± standard deviation (SD). Mann-Whitney U tests confirmed significant differences between each pair of methods (p<0.01). The FID results matched the overall image quality scores in the expert reader study. The proposed SPTSR method achieved the best FID scores. This quantitative analysis confirmed the proposed method has the best image visual quality.

#### 4) ISOTROPIC SR RESULTS

**FIGURE 8.**
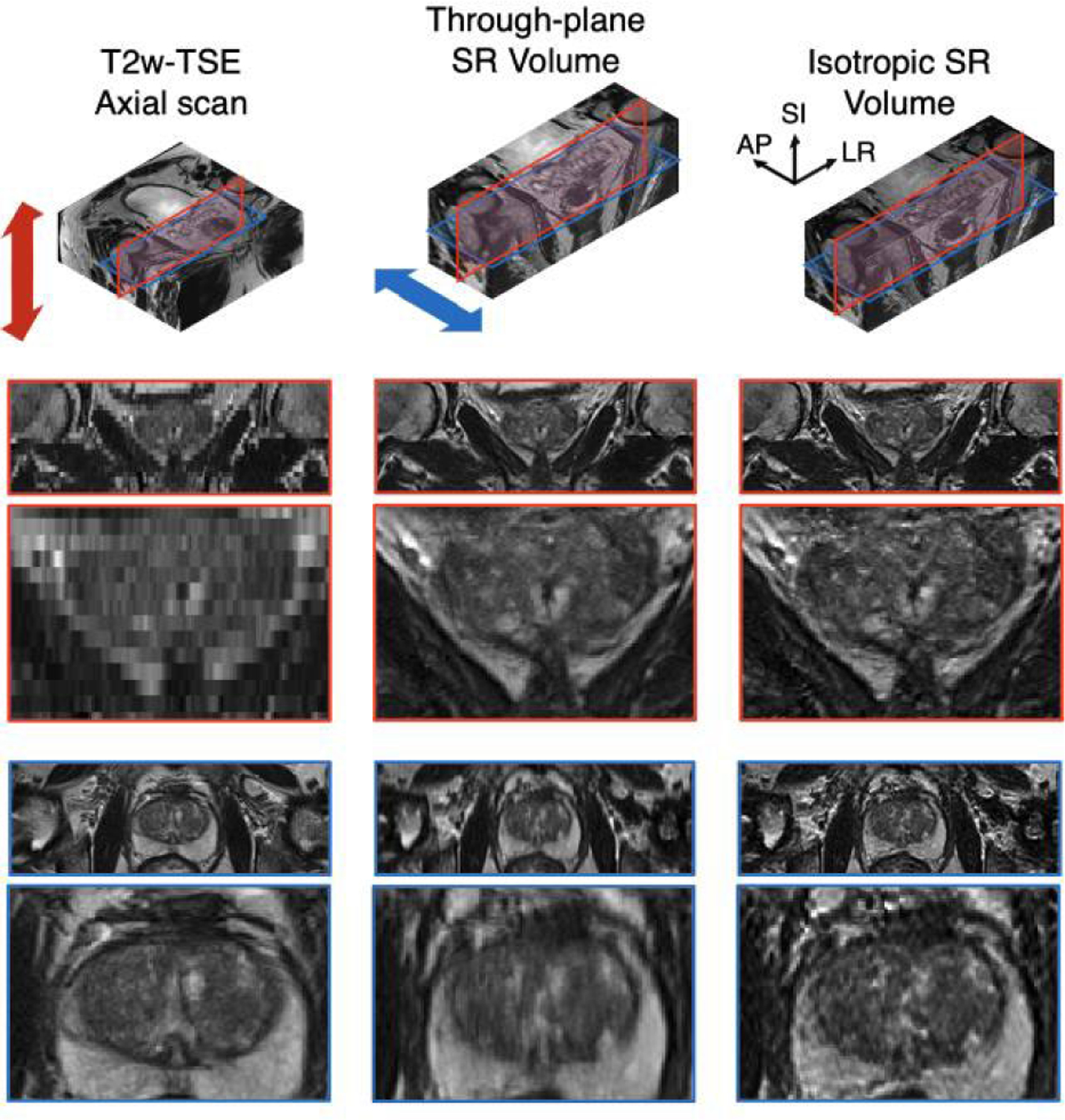
The isotropic SR output (right), compared to the original T2w-TSE axial scan input (left) and through-plane SR volume (middle) with elongated voxel. Two-sided arrows indicate the orientations of the slice-profile PSF as the blur kernel. Red patches represent coronal views and blue patches represent axial views.

Fig. 8. shows the SP-deconvolved isotropic SR results from the through-plane SR image volume. The through-plane results have convolved by the same slice-profile PSF of the orthogonal axial scan, so even though it has an isotropic voxel spacing, its image in the original coronal plane is blurred by the slice-profile and does not retain its sharpness, compared to the coronal scan input. After the SP-deconv via Richardson-Lucy deconvolution in the AP-direction [40], [41], the blurring artifacts in the coronal plane are deblurred, resulting in an isotropic image volume with non-overlapping cubic voxels.

### B. 3D SIMULATION EXPERIMENTS

#### 1) ISOTROPIC SR RESULTS

**FIGURE 9.**
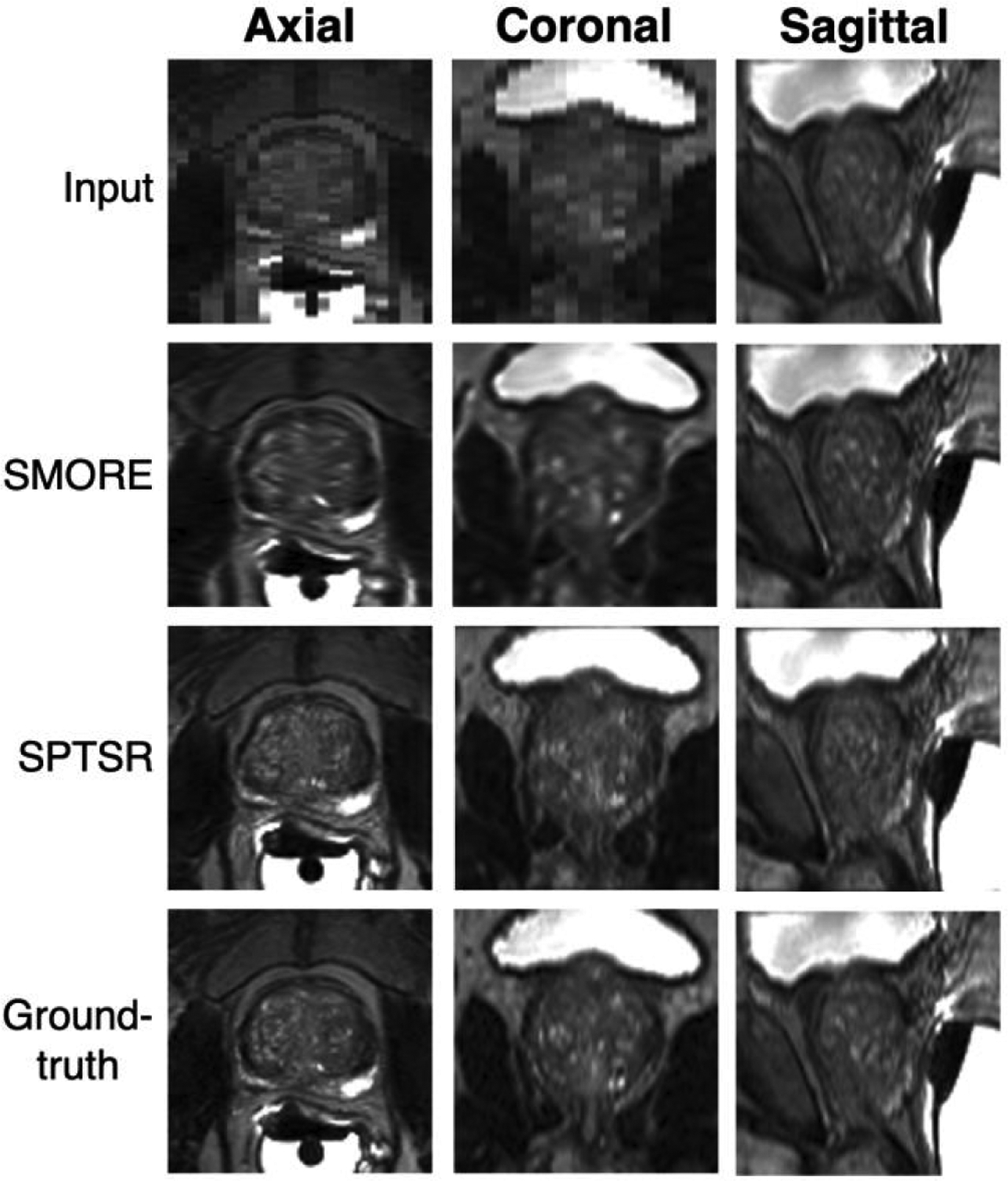
Simulation experiments result with 3D T2w scans. Simulated 2D sagittal input, SMORE result, SPTSR result were compared with isotropic high-resolution ground-truth images in all three views.

The simulated 2D sagittal scans input, SMORE and SPTSR results were compared against the isotropic 3D T2w ground-truth in all three orientations, as shown in Fig. 9. The images from SMORE were noticeably blurry in the super-resolved coronal and axial views, while the images from SPTSR considerably match well with the 3D T2w ground-truth in all three views.

#### 2) QUANTITATIVE ANALYSIS

The PSNR and NMSE measurements for simulation experiment are shown in Table 4. For each method, the PSNR and NMSE of 118 3D volumes are shown as mean ± SD. Paired samples t-test confirmed significant differences (p<0.01) between each pair of methods for both PSNR and NMSE.

The numerical results indicated that the proposed SPTSR method can super-resolve to isotropic high-resolution volume, and performs significantly better than SMORE when super-resolving images to isotropic high-resolution volume. This result also indicated that the final SP-deconvolution step is necessary to achieve high-quality isotropic super-resolution.

### C. ABLATION STUDIES

#### 1) INDIVIDUAL CONTRIBUTIONS FROM TWO IMPROVEMENTS

**FIGURE 10.**
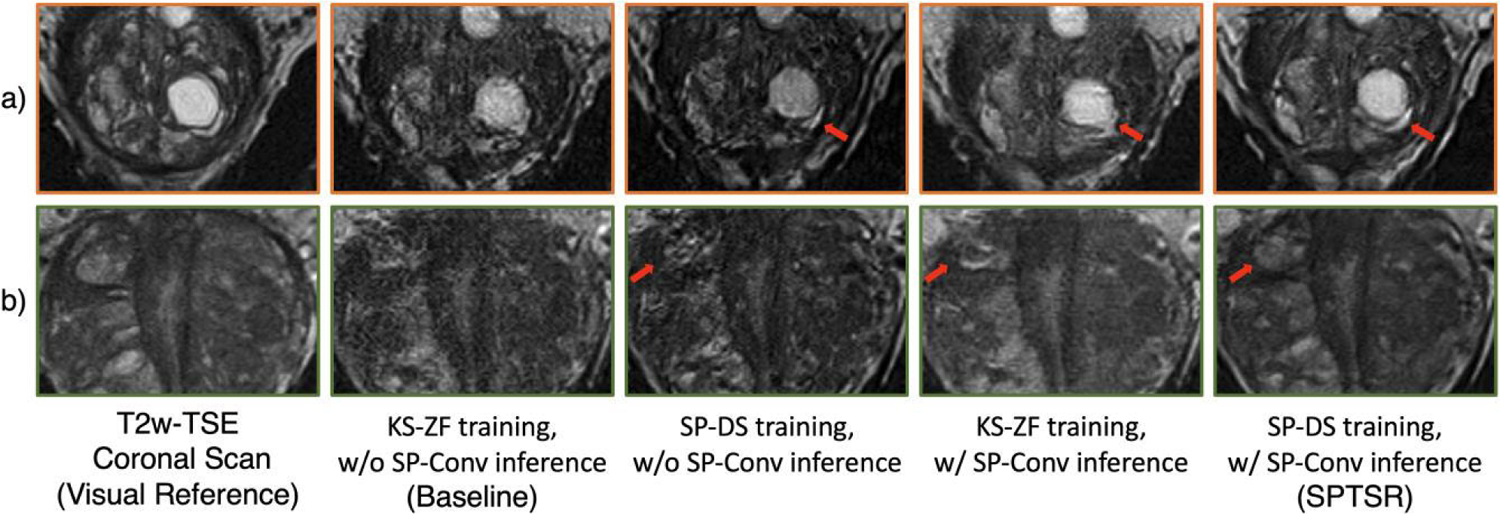
The individual impact of SP-DS Network and SP-Conv input on testing output. a) and b) represents images from two different testing subjects, same as Fig. 6. From left to right: The T2w coronal scan of the subject, as a visual reference; the baseline KS-ZF downsampling method trained network, without SP-Convolved inference input; SP-DS method trained network, without SP-Convolved inference input; KS-ZF downsampling method trained network, with SP-Convolved inference input; the proposed SPTSR inference output with SP-DS method trained network, and SP-convoluted inference input. Red arrows indicate the structural differences between different results.

To compare the separate contributions from the SP-downsampled input trained network and the SP-convolution pre-processed inference input, four methods were compared:

1. KS-ZF coronal scan training data, without SP-conv pre-processed axial scan inference testing data;
2. SP-DS coronal scan training data, without SP-conv pre-processed axial scan inference testing data;
3. KS-ZF coronal scan training data, with SP-conv pre-processed axial scan inference testing data;
4. SP-DS coronal scan training data, with SP-conv pre-processed axial scan inference testing data (Proposed);

The comparison results are as shown in Fig. 10. Without either SP-downsampled training input or the SP-conv pre-processed inference input, the inference results did not achieve the desired sharpness and noise level, and could not fully recover the structural details.

#### 2) NETWORK ARCHITECTURES

The networks architectures ablation study is presented in Table 5. We compared three popular network architectures to our proposed networks. In terms of quantitative image metrics in the validation dataset, our proposed architecture achieved the best mean PSNR of 24.64, and the best mean SSIM of 0.817. The PSNR and SSIM were close compared to the U-Net and ResUNet architecture, but those two networks are significantly larger in the number of parameters. Our comparison with U-Net and ResUNet demonstrates that our proposed SPTSR does not need a large network to achieve excellent SR results. On the other hand, SRGAN has approximately the same number of parameters as our proposed network, but SRGAN has the worst PSNR and SSIM numbers among all the networks. Compared to SRGAN, the main improvement from our proposed network is the use of 3-slice input instead of SISR in the SRGAN structure. Because of the SP-convolved input, the image voxels overlap in the slice direction. The use of 3-slice input borrows image information from the adjacent slice, which not only helps the through-plane SR results but also preserves the inter-slice consistency across the image volume and benefits the isotropic SR results.

#### 3) SIZE OF THE TRAINING DATASET

**FIGURE 11.**
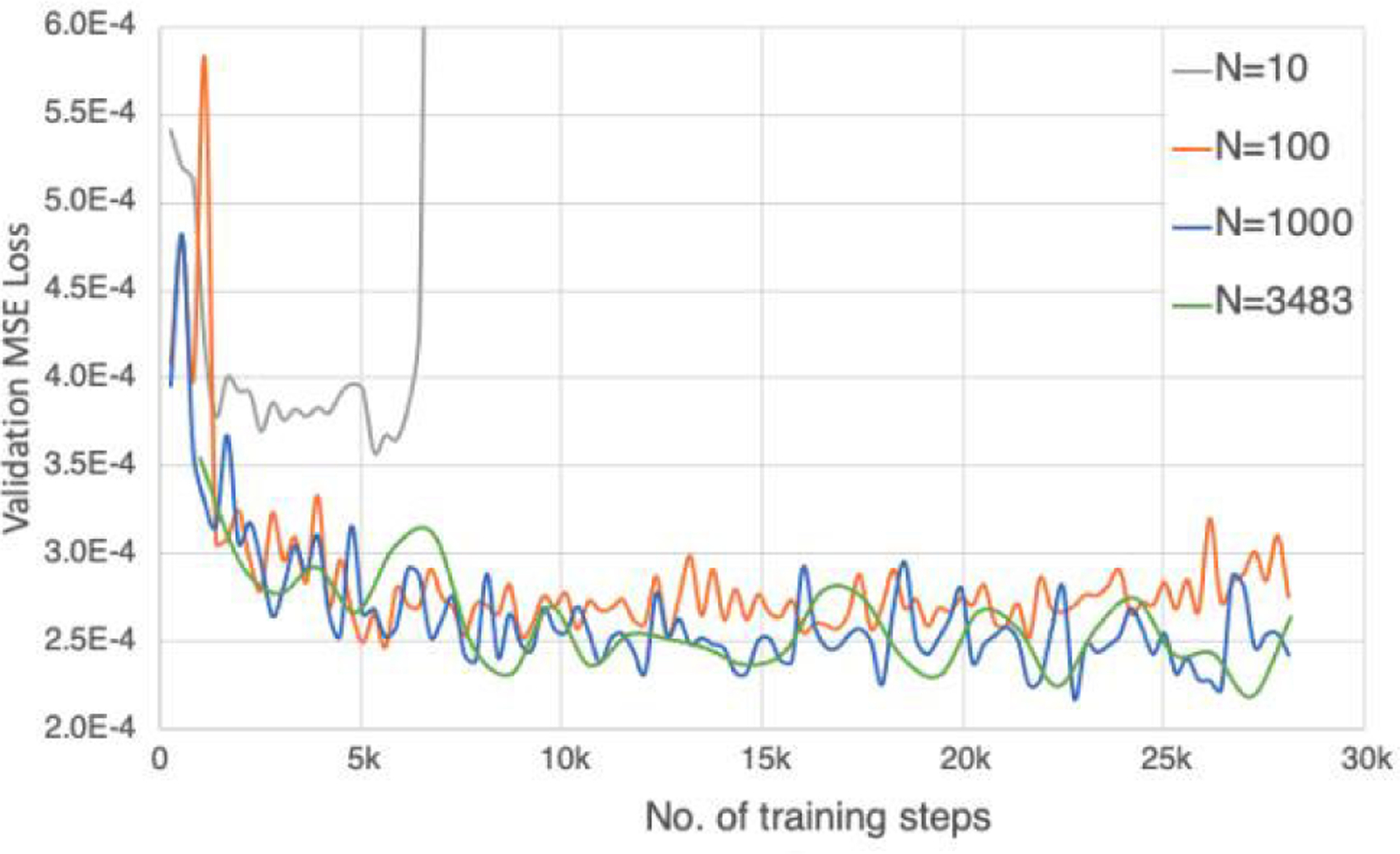
Validation MSE loss with different sizes of the training dataset. The number of training dataset cases are 10, 100, 1000, and 3483 respectively. The MSE loss on the validation dataset is plotted against the number of training steps, with the same batch size.

The validation MSE loss versus the number of training steps for different sizes of the training dataset is plotted in Figure 11. When N=10, the training dataset was too small, and the networks training was quickly overfitted, as the validation MSE loss start to skyrocket after 5k training steps. The validation MSE loss plots were similar for N=100, 1000, and 3483. This indicates the size of training dataset is sufficient on the order of 100 subjects. This is mainly because our proposed networks used a relatively small number of parameters, as shown in Table 5.

#### 4) SLICE PROFILES

The slice profiles ablation study is presented in Table 6. Training and validating with datasets downsampled by truncated sinc produced better SSIM and PSNR results than other combinations, but all four comparisons showed no significant differences. Training and validating with Gaussian PSF had similar SSIM and PSNR compared to training and validating with truncated sinc PSF, indicating a slight inaccurate slice profile used does not affect the image quality; Training and validating with different slice profiles also had similar SSIM and PSNR, indicating retraining may not be required when the dataset included with different slice profiles was used.

## V. DISCUSSIONS

We proposed a novel slice-profile transformation-based super-resolution (SPTSR) framework for multi-slice 2D TSE MRI. We utilized a large 2D/3D dataset of clinical subjects and scans and demonstrated the visual improvements for 5.5x through-plane SSR compared to the k-space zero-filling-based baseline method and the SMORE method. We also conducted 3D simulation experiments to demonstrate visual and quantitative improvements for 4x isotropic SSR compared to the 3D T2w ground-truth.

The testing output images for 2D clinical scans experiment were compared to the SMORE method [26], and a baseline KS-ZF trained network method. The output image quality was qualitatively evaluated on a 4-point Likert scale by two genitourinary radiologists in a blinded study. Quantitative analysis with FID was conducted to cross-validate with the reader study.

The testing output images for 3D simulation experiment demonstrated the visual improvement for SPTSR method compared to the SMORE method. The quantitative results of PSNR and NMSE confirmed the superiority of SPTSR. The quantitative comparison between before and after the last SP-deconvolution step indicated the effectiveness of the SP-deconvolution when achieving isotropic super-resolution.

The network structure ablation study justified our network structures and confirmed our SPTSR framework to be model-agnostic. The training dataset size ablation study confirmed that 2,000 images from 100 subjects were sufficient for network training because the proposed networks used a relatively small number of parameters. The slice-profile ablation study confirmed that simulated slice profiles are sufficient for the SPTSR framework. Furthermore, we demonstrated the preliminary results of deconvolved isotropic SR from the through-plane SR image volumes.

In previous studies of super-resolution reconstruction (SSR), most approaches use multiple 2D scans, whereas our method only uses a single 2D scan for super-resolution. In previous studies for deep-learning-based super-resolution, most approaches consider the synthesis of LR images a single-image problem, opting for the k-space zero-fill or interpolation/averaging method [14], [15], [16], [17], [18], [19], [21], [22], [23], [24]. Our SPTSR method takes the slice profiles of both training and testing scans into consideration, jointly bridging the physical differences between training and testing data.

Compared to the baseline methods of using a KS-ZF trained network for the inference of orthogonal volumes, our proposed SPTSR methods output much sharper, less artifact, and much less noisy SR images. This is because there exist fundamental differences between training and testing data as the conventional approaches did not take slice profiles into consideration. Our proposed method bridges the gap between the two by taking both slice profiles into consideration. The image results are strongly supported by our reader study results. The proposed SPTSR method received excellent overall image quality for 47 out of 50 cases, significantly better overall image quality than other methods (p<0.01). The quantitative analysis of FID with the testing dataset also confirmed the superiority in perceptual image quality of the proposed SPTSR method. This shows the effectiveness of SPTSR and the feasibility of replacing multiple multi-slice 2D MRI scans with a single multi-slice 2D scan.

The proposed method combined SP-DS trained network and SP-convolution pre-processing to improve the overall image quality. Only changing the network from the baseline KS-ZF trained network to SP-DS trained network resulted in a sharper but noisier image compared to the proposed method. This is because the training input voxel is blurred by the slice-profile kernel in two orthogonal directions, whereas the inference input without SP-conv is blurred in only one direction. Only pre-processing the inference input with SP-convolution resulted in a smoother image, lacking sharpness and contrast. This is because the KS-ZF downsampled image is physically different from the inference input image.

Our method can be applicable to a wide range of MRI applications since multi-slice 2D MRI sequences are used in many different applications, including knee, prostate, brain, placenta, and fetal brain [3], [5], [7], [29], [52]. Because our method focuses on improving the training input synthesis and inference input pre-processing, our method would not be limited to specific deep-learning architectures or training schemes. Thus, novel deep-learning method advancements can be applied jointly in future studies.

Our study in this paper included several limitations. First, current network training only considers the loss functions in through-plane SR. Deep-learning-based deconvolution methods [53], [54] enable self-consistency losses in the in-plane orientation, which can help further improve isotropic SR output. Second, our current approach does not account for inter-slice motion artifacts. In some applications, such as fetal brain MRI, motion is obvious and unavoidable [29], while patient motion during a typical prostate MRI scan is less significant [55], [56]. A more motion-robust SR framework can be achieved by including simulated motion in the training data, or enforcing a regularization loss term in the through-plane direction.

## VI. CONCLUSION

In conclusion, we developed a novel slice-profile-transformation-based super-resolution (SPTSR) framework for super-resolution of multi-slice 2D MRI scans. The proposed slice-profile transformations bridge the inherent physical mismatches between training and testing inputs due to an imperfect slice-selection profile. A large set of 4,878 pairs of axial and coronal MRI scans were used for training, validation, and testing of the proposed SPTSR framework. The expert reader study and experimental validation demonstrated the effectiveness of SPTSR in 5.5x through-plane SR with isotropic voxel spacing. Furthermore, we illustrated that SPTSR can achieve the isotropic SR with non-overlapping cubic voxels with the 3D simulation experiment.

## Figures and Tables

**TABLE 1.** The T2-weighted turbo spin-echo (T2w-TSE) sequence parameters.

TR/TE (ms)	4000/101
Refocusing angle (degree)	160
Bandwidth (Hz/pixel)	200
Acquisition matrix size	320×320
In-plane resolution (mm^2^)	0.625×0.625
Number of slices	20
Slice thickness (mm)	3.0
Slice spacing (mm)	3.6
Number of averages	2
Scan time (mm:ss)	01:40×2

**TABLE 2.** The 3D T2-weighted SPACE sequence parameters.

TR/TE (ms)	2200/201
Refocusing angle (degree)	100
Bandwidth (Hz/pixel)	315
Acquisition matrix size	256×256×60
In-plane resolution (mm^2^)	0.664×0.664
Slice thickness (mm)	1.5
Scan time (mm:ss)	07:00

**TABLE 3.** Testing quantitative results with FID.

Bilinear Interpolation	32.0 ± 12.5
SMORE [26]	29.5 ± 14.8
K-space Zero-fill	26.8 ± 10.4
SPTSR	**18.6** ± **8.5**

**TABLE 4.** Simulation testing quantitative results with PSNR and NMSE.

	PSNR	NMSE (×10^−2^)
SMORE [26]	26.81 ± 3.03	2.00 ± 1.15
Before SP-deconv	28.82 ± 2.90	1.00 ± 0.68
SPTSR	**29.08** ± **2.92**	**0.92** ± **0.54**

**TABLE 5.** The network structure ablation study validation results comparison.

	Number of Parameters	SSIM	PSNR
U-Net [50]	17.3M	0.811±0.033	24.48±1.66
ResUNet[51]	13.0M	0.797±0.041	24.41±1.62
SRGAN [38]	**0.5M**	0.808±0.032	24.30±1.73
SPTSR	**0.5M**	**0.817±0.031**	**24.64±1.67**

**TABLE 6.** The slice profiles ablation study with gaussian PSF and truncated Sinc PSF.

	SSIM	PSNR
Trained w/ Gaussian, validated w/ Gaussian	0.804±0.033	24.18±1.68
Trained w/ Gaussian, validated w/ trunc sine	0.806±0.033	24.29±1.64
Trained w/ trunc sine, validated w/ Gaussian	0.804±0.033	23.94±1.71
Trained w/ trunc sine, validated w/ trunc sine	**0.817±0.031**	**24.64±1.67**
